# Uncovering the Effects of COVID-19 Mask Wearing on Bird Flight Initiation Distance in Urbanized Areas in the Southern Philippines

**DOI:** 10.3390/ani13081289

**Published:** 2023-04-09

**Authors:** Gerald Vince N. Fabrero, Leanne Jay S. Manceras, Angelo Rellama Agduma, Krizler Cejuela Tanalgo

**Affiliations:** 1 Ecology and Conservation Research Laboratory (Eco/Con Lab), Department of Biological Sciences, College of Science and Mathematics, University of Southern Mindanao, Kabacan 9407, Cotabato, Philippines; gvnfabrero@usm.edu.ph (G.V.N.F.); geloagduma@gmail.com (A.R.A.); 2 Department of Biological Sciences, College of Science and Mathematics, University of Southern Mindanao, Kabacan 9407, Cotabato, Philippines; 3 State Key Laboratory for Conservation and Utilization of Subtropical Agrobioresources, Guangxi University, Nanning 530004, China; 4 Guangxi Key Laboratory of Forest Ecology and Conservation, College of Forestry, Guangxi University, Nanning 530004, China

**Keywords:** anthropause, bird escape strategies, COVID-19, green spaces, environment, Philippines, urbanization

## Abstract

**Simple Summary:**

The COVID-19 pandemic has caused changes in human behavior, such as wearing masks and reducing public activities, which have also affected the behavior of urban wildlife. However, the impact of COVID-19-related activities, particularly mask wearing, on urban wildlife is not yet fully understood, especially in the Philippines, where COVID-19 mask wearing restrictions persisted longer than in other nations. To address this, we assessed the response to mask wearing of two common urban bird species by examining their flight escape strategies, such as alert and flight initiation distances. Our study showed that mask wearing reduces bird vigilance against intruders, but the response was species-specific.

**Abstract:**

The COVID-19 pandemic has caused significant changes in public and human activities worldwide, including using masks and reducing human interaction. These changes have also affected wildlife behavior, especially in urban areas. However, there is limited understanding of the impact of COVID-19-related human activities, mainly mask wearing, on the behavior of urban bird species. This case is intriguing in the Philippines, where COVID-19 restrictions and mask wearing have been more prolonged than in other countries. We studied two common urban bird species (*Geopelia striata* and *Passer montanus*) in Southcentral Mindanao, Philippines, to assess their response to mask wearing by examining their alert distance (AD) and flight initiation distance (FID). We found that birds had a reduced FID to mask wearing, but only significantly in *G. striata* (Zebra Doves) and not in *P. montanus* (Eurasian tree sparrow). The effect of the variables related to urbanization on FID was contrasting. For example, ambient noise increased bird vigilance while proximity to roads reduced bird FID in urbanized areas, but their effects were weaker compared to mask wearing. We conclude that mask wearing during the COVID-19 pandemic is a significant environmental element that alters bird escape responses in urban areas, and the effects may be species-specific.

## 1. Introduction

The COVID-19 pandemic began to spread at the end of 2019 [1,2], which affected over 210 countries [3]. The public was advised to wear facemasks and limit mobility as part of public health interventions to prevent the increase of COVID-19 infection. These public safety measures lasted two years and have reduced human movement and activities. Scientists describe it as ‘anthropause’ [4,5,6] that resulted in significant changes in the ecosystem, including reducing air and water pollution [7,8], lowering the mortality of road animals, and improving the physiological condition and reproductive success of animals [9]. Furthermore, since pandemic lockdowns substantially reduced the overall human activities, there are consequent temporary increases in animal movement in urban areas [10,11,12]. At least 80% of focal birds in the United States were observed to alter and use more urban areas compared to the pre-pandemic period, indicating that birds benefited from lockdowns [13]. Furthermore, while specific reports were anecdotal, such as the observation of increased dolphin activity in the Bosphorus Strait, one of the world’s busiest maritime channels in Istanbul, Turkey, possibly caused by traffic halts and fishermen staying home due to the city lockdown [14,15]. These observations have allowed researchers to investigate how animals react to rapid environmental changes caused by human activities during the COVID-19 pandemic [6,12,16].

The presence of a predator influences animal behavior and causes the evolution of a diverse spectrum of anti-predator behavior throughout the animal kingdom [17]. For example, cave-dwelling bats cluster together in larger colonies to reduce crow predation [18], while birds use alarm calls to alert conspecifics of the presence of a predator [19]. The prey–predation relationship varies when new environmental factors are introduced, and animals are expected to cope with these new features; however, this is often specific to the species, habitat conditions, and types of disturbance present [13,20,21,22]. For example, trees provide birds with a location to perch, reducing the energy spent fleeing intruders or predators [23,24]. When birds feel safe in trees or potential nesting grounds, they are more willing to tolerate disturbance near potential terrestrial predators or human intruders [24,25]. The investigation of animal escape strategies is widely studied among birds because they are widespread and can develop rapid responses. They are sensitive to their surroundings and are constantly exposed to noise, particularly in urban areas [25,26,27]. 

Studying the flight initiation distance (FID) allows us to know how animals strategize and develop their escape from potential threats or predators within specific habitat types [23,26,28]. Throughout the COVID-19 pandemic, studies have shown that birds have become less cautious of humans [13,29], demonstrating how quickly they can alter their responses to new environmental features and tolerate urban areas [10,29]. Intruders and their elements can influence the distance at which animals will initiate their escape [30,31]. 

In addition, various studies have demonstrated the effects of COVID-19-related activities on changes in animal behavior. For instance, Jiang et al. found that mask wearing decreased bird vigilance toward individuals wearing masks [29]. In contrast, the opposite was observed for Nubian Ibex, showing increased vigilance in response to mask wearing [9]. However, understanding the effects of COVID-19-related human activities, particularly mask wearing, on the behavior of urban wildlife, remains limited [10,29]. This case is particularly interesting in the Philippines, where the COVID-19 restrictions and mask wearing were more prolonged (i.e., mandatory mask wearing was only lifted in October 2022, after we conducted this study) compared to other countries [32]. 

In this study, we report the first investigation in the Philippines on the escape strategy of urban birds in response to mask wearing during the COVID-19 pandemic. Our study used two focal species of urban birds: the Eurasian tree sparrow (*Passer montanus*) and the zebra dove (*Geopelia striata*). Due to their widespread distribution, they are common in all urban areas of the Philippines [33,34] making them ideal candidates for our investigation. 

## 2. Materials and Methods

### 2.1. Study Sites and Target Species

We conducted our field observation in the urbanized zones of Kabacan (7.107° N, 124.840° E) and Carmen (9.840° N, 124.198° E) in the Cotabato province, in Southcentral Mindanao, Philippines, from July to October 2022. All target study sites have similar structures and conditions with commercial and residential infrastructure with fragments of tree cover and are surrounded by agricultural land. The identified study sites were characterized as frequently visited by humans and near human settlements.

Our study focused on the escape strategy of two cosmopolitan urban bird species in Southcentral Mindanao, namely, the Eurasian tree sparrow (*P. montanus*) and the Zebra dove (*G. striata*) [34,35]. The Eurasian tree sparrow is a small (14–15 cm in body length) and diurnal urban species, characterized by its black patch with a pure white cheek, chestnut crown, and nape. They mainly forage on the ground for seeds and small invertebrates in urban areas. Zebra doves are characterized by a long tail, mostly brownish-gray in color, with black and white barring. They are distinguished by their pleasant, soft, staccato cooing. They grow to a length of 23 to 27 cm. Zebra doves forage on bare ground and short grasses, and feed on small grasses, seeds, and small invertebrates. These two species of birds are abundant and widespread throughout the Philippines, and are highly tolerant of urbanization and disturbances [36].

### 2.2. Bird Alert Distance (AD) and Flight Initiation Distance (FID)

**Figure 1:**
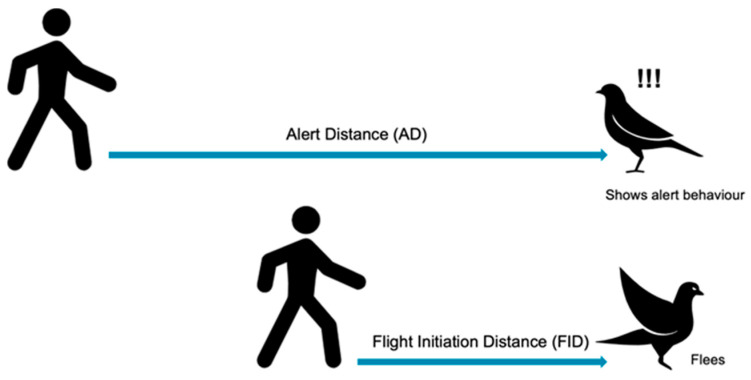
Graphical representation showing the bird alert distance (AD) and flight initiation distance (FID).

We measured the individual bird FID using modified methods from recent studies [24,29]. We began our observation after the bird had been sighted on the ground, at least 10 m from the observer/intruder. The starting distance ((SD), the distance from the intruder to the focal bird when first spotted and the FID trial commenced) was marked using a colored clay ball and measured using Mileseey Laser Range Finder™ (Mileseey, Shenzhen, China), with a range capacity of 100 m. After marking the SD, the intruder approached the target bird at a constant average walking speed (~1 m/s), keeping eye contact while heading straight to the target bird. We measured the bird alert distance (AD) or the distance between the intruder and the individual or group of birds detecting threats, for example, head-turning around or moving further away, but without fleeing. Then we measured the flight initiation distance (FID) based on the distance at which the bird fled or escaped as a stimulus response to the intruder (Figure 1).

**Figure 2:**
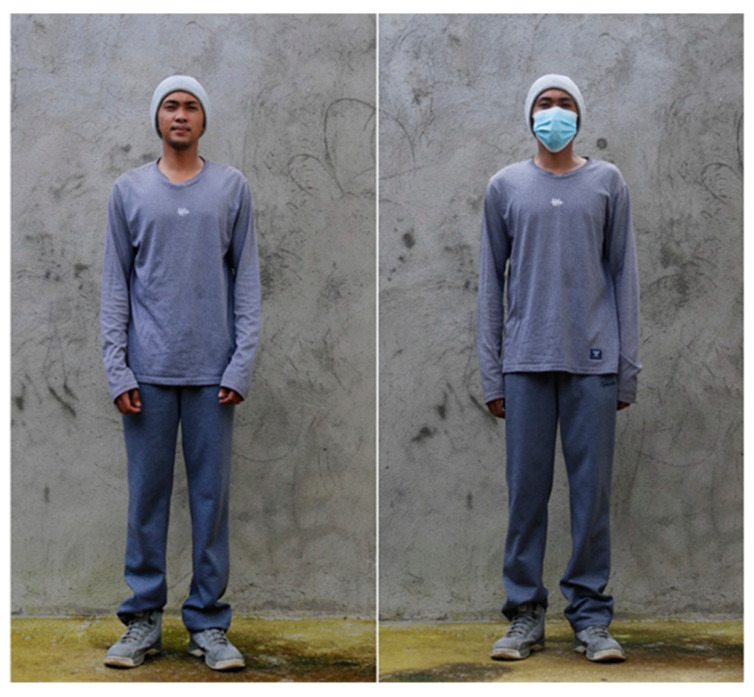
Intrusion treatment (**left**: without the mask on, **right**: with the mask on) and neutral clothing of the intruder. Photographs by: G.N. Fabrero.

We set up two observation treatments to determine the effects of mask wearing on birds: (i) mask wearing (blue surgical mask) and (ii) non-mask wearing. To reduce bias in the behavioral response, observers/intruders for mask and non-mask treatments were the same person (VGNF), who wore neutral clothing without body accessories and, who was trained to walk at a constant pace of ~1 m/s (Figure 2). We only considered an individual or group of birds observed at the ground level, since a vertical position (e.g., perching on trees or human structures) can affect bird detection of the intruder and influence the FID [21,29]. We selected a single focal individual bird for observation when birds occurred in flocks. Birds were observed opportunistically at different locations to maximize the sampling size. Field observations were consistently done in the morning, from 06:00 AM to 10:00 AM, or predusk from 04:00 PM to 06:00 PM, where birds are increasingly active due to cooler weather. We avoided data collection during rainy weather. Furthermore, to reduce bias, we did not sample on the same day within at least 50 m from the area of previous observations. Overall, we made 289 attempts, and only 148 were successful. 

### 2.3. Statistical Analysis and Modeling

In our final analysis, we only modeled bird flight initiation distance (FID) as we initially found a high correlation between AD and FID (Pearson’s *r* = 0.774, *p* = 0.001). In addition, the initial analysis also showed no significant difference in bird FID during the morning or afternoon (MWU test = 2163, *p =* 0.136), so we excluded this as a factor in our final analysis. As our dependent variable did not conform to the data normality assumptions, we rounded our FID values to integers. We conducted a comprehensive generalized linear model (GLM) with a Poisson error distribution, utilizing a log-link function, to explore the association between bird flight initiation distance (FID) and various predictors. We included mask wearing, bird species, starting distance, flock size, ambient noise, and distance to roads as our explanatory variables. We determined the flock size of the species by counting the number of individuals observed within a 10 m radius of the focal species. Ambient noise (dB, decibels) was measured three times from the starting distance (SD) to the flight initiation distance (FID) using the Bside noise tester (TA8151) (Shenzhen Bside Trade Co., Ltd. Test & Measurement, Shenzhen, China). From the spot where we recorded the FID, we calculated the nearest distance to the roads and included them as independent variables. A separate GLM per bird species was performed for the FID to determine species-specific responses to the same independent variables. 

To model and visualize bird FIDs, we used the *Gamlj* module in the open software JAMOVI 2.3.19. [37,38] and performed all spatial analyses in QGIS 3.16 [39]. We set the significance level at *p* < 0.05.

### 2.4. Ethical Notes

We did not collect any animals during the study period. We adhered to the Animal Behavior Society (ABS) and the Association for the Study of Animal Behavior (ASAB) guidelines for the treatment of animals in behavioral research and teaching [40]. 

## 3. Results

**Figure 3:**
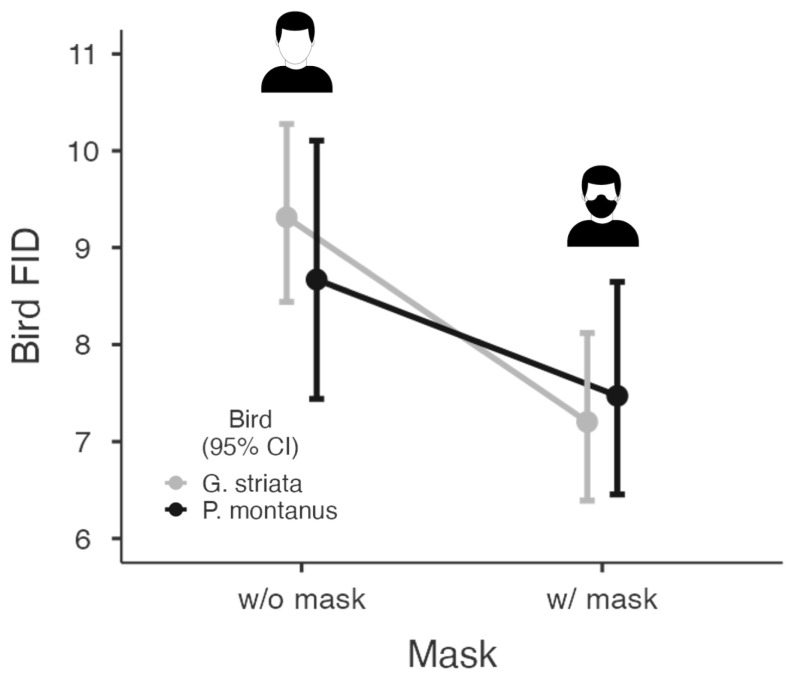
Visualized results of the GLM showing the effects of mask wearing on flight initiation distances of two bird species (*G. striata* and *P. montanus*). Whiskers indicate 95% CI.

During the survey period, we had 148 observations (*G. striata*: *n* = 93; *P. montanus*: *n* = 55). Our observations revealed that wearing masks typically reduced the bird flight initiation distance (FID), although the degree of response varied between the two species (Figure 3). We found that the FID was not affected by bird species (MODEL A), but mask wearing reduced the bird FID (β = −1.794, *p* = 0.011). We found a significantly shorter FID in birds approached by the intruder with a mask (6.85 ± 3.50) compared to birds approached by the intruder without a mask (10.82 ± 6.818). The increase in starting distance (SD) also increased the bird FID (β = 0.496, *p* < 0.001). Among environmental variables, ambient noise (β = 0.088, *p* = 0.021) and distance to the road (β = 0.032, *p* = 0.037) positively affected the overall increase in the bird FID (Table 1). We then separately evaluated the response of the two species and found that mask wearing had significantly reduced the FID of *G. striata* (MODEL B; β = −2.543, *p* = 0.006) and not of *P. montanus* (MODEL C; β = −1.223, *p* = 0.221). However, SD significantly affected FID for both species models (Table 1). Contrary to our expectations, the size of the flock did not have a significant effect on the FID.

## 4. Discussion

In a rapidly developing urbanized environment, animals adjust their responses to stimuli to co-exist with humans, but their responses to new elements vary with species and habitat types [9,20]. The COVID-19 pandemic has affected human movement and animal interactions with the environment [13,16]. Therefore, it is important to understand changes in animal responses, particularly how they respond and acclimate to their environment with the new element. Here, we assumed that changes in human activities (mask wearing) during the pandemic altered bird species’ responses, particularly in urbanized areas of the Philippines, where mask wearing and lockdown periods were more prolonged than in other countries [32].

Our study revealed that the bird FID was significantly influenced by mask wearing and that the response levels vary by species. We found that mask wearing significantly reduced the FID of *G. striata* only. *Passer montanus,* on the other hand, did not respond to the new environmental element. This means that in urbanized habitats, *G. striata* is more sensitive to mask wearing compared to *P. montanus*. One plausible explanation is that *P. montanus* adapted more quickly to the new environmental elements and was less sensitive to mask wearing than *G. striata*. In a previous study by Jiang et al. [29], mask wearing decreased the FID of urban sparrows (*Passer* sp.) in China. However, they stressed that altered bird escape strategies might revert to pre-pandemic levels once adapted to new environmental elements or when normal activities resume. The adaptive capacity of Eurasian sparrows and their higher cognitive learning may have aided their response to changes induced by humans [41], such as mask wearing. Notably, a large-scale bird FID study conducted by Mikula et al. [10] in Europe showed that mask wearing is not strongly associated with the alertness and vigilance of multiple species of birds on the continent.

Our observation found a strong relationship between the FID and SD for both species and mask wearing treatments. The positive association of SD with FID is widely observed in many studies and suggests that SD is an important factor in the variability of FID between species [28,30]. The escape theory may explain the relationship we observed, which indicates that the prey makes an escape decision according to its ability to perceive a threat [23,28,30]. Therefore, if monitoring becomes costly, e.g., the farther SD, the prey must detect any approaching intruder as early as possible and consider them a potential threat [23,42,43]. This relationship further suggests that our sampled birds assessed the risk of intrusion at a greater distance, regardless of whether the intruder was wearing a mask.

Furthermore, we expected that the flock size would affect the bird FID [24,29], but we found otherwise. However, it is interesting that our sampled bird species have contrasting responses to flock size. For example, the size of the flock positively affected the FID of *P. montanus,* but had a negative effect on the FID of *G. striata.* The larger flock formation may explain this in *P. montanus* compared to *G. striata*, which often occurs singly or in pairs [35]. The flock size of animals is positively related to the early warning communication strategy of the individuals in the group, especially with highly social species [24]. This holds particularly true for larger flock sizes in *P. montanus*, as the presence of multiple eyes enables the species to perceive potential threats from various directions. Consequently, this allows the species to effectively identify and defend themselves against any potential predators or intruders, as the awareness of multiple individuals is combined to form a collective response [24,44,45]. 

We hypothesized that, in addition to mask wearing, urbanization influences the responses of birds to intruders [13]. We found that the FID of birds increased farther they were from the roads. This observation suggests that birds residing in less disturbed areas exhibit higher vigilance toward potential intruders. These findings are similar to a recent study in Beijing, which showed that the bird FID is lower in areas with high urbanization than in more pristine habitats (i.e., lesser roads) [46]. Xu et al. [47] also showed that the road distance increased with the bird FID, while the FID of snow finches in Qinghai-Tibet, near the railway and highway, was significantly reduced [48]. In addition, we also found that the bird FID was positively correlated with ambient noise levels. However, previous investigations showed that traffic noise is not a primary factor in altering bird activity near highways, but is mainly attributed to birds evading traffic-related mortality [49]. Our current findings suggest the sampled birds perceived noise as an indication of the presence of a potential intruder, which can trigger a response in birds to become more vigilant and alert [27,50]. In addition, birds may perceive the disturbance as a more significant threat and need more time to assess the situation before taking flight.

The ability of bird species to adjust their flight initiation distance (FID) and tolerance to urbanization often depends on the availability of resources in the surrounding area and their ability to adapt to new features [49,51]. Birds in urban areas tolerate and adapt to living close to humans, to take advantage of the resources available in urban areas to find food at the expense of their vulnerability to predation [24,52,53,54]. However, the use of face masks, being a new and unfamiliar element, may have been perceived as a more significant threat than urbanization itself, which is evident from the greater effects it had on bird vigilance compared to variables related to urbanization. In another case of mask wearing effects, evidence from Yosef et al. [9] showed an opposite response from Nubian ibex, and its vigilance increased to intruders that wore anti-COVID-19 masks. Our findings and previous observations suggest that animal responses to mask wearing during the pandemic vary widely and are species-specific.

Urban environments are expected to promote greater tolerance of birds to humans, because they face a lower risk of predation than rural birds [55,56]. The preference of sparrows for areas where humans wearing masks are more frequent could explain their faster adjustment (i.e., reduced FID) to mask wearing. Previous studies have shown that house sparrows residing in urban areas become more habituated to human activities (e.g., reduced fear and vigilance) more quickly than their rural counterparts [57]. While Zebra doves prefer less disturbed environments when foraging [34], they probably require a more extended period to adjust to the new environmental element. We propose that mask wearing is a significant environmental element that could alter bird responses in urban areas. However, the extent of its effects may vary depending on the species. It is crucial to recognize that our interpretation of the results may be constrained by the absence of evidence from the pre-pandemic period, and should thus be taken with care. More experiments in the post-pandemic period should be explored to further understand the impact of mask wearing on bird responses.

## Figures and Tables

**Table 1:** Results of the generalized linear model (GLM) to determine the relationship between bird FID of all species (A), *G. striata* (B), and *P. montanus* (C) to mask wearing, species traits, and urbanization variables. The variables in bold indicate significance.

(A) All Bird Models			
**Variables**	**Estimate (β)**	**SE**	***p***
(Intercept)	2.094	0.032	<0.001
**w/mask-w/o mask**	**−0.203**	**0.066**	**0.002**
*P. montanus-G.* *striata*	−0.018	0.075	0.816
mask ✻ bird species	0.108	0.125	0.387
Flock size	0.011	0.012	0.359
**SD (m)**	**0.047**	**0.004**	**<0.001**
**Ambient noise (dcb)**	**0.007**	**0.003**	**0.040**
**Distance to roads**	**0.004**	**0.001**	**0.008**
**(B) *Geopelia striata***			
(Intercept)	2.199	0.036	<0.001
**w/mask-w/o mask**	**−0.287**	**0.078**	**<0.001**
Flock size	−0.017	0.024	0.483
**SD (m)**	**0.046**	**0.004**	**<0.001**
Ambient noise (dcb)	0.006	0.004	0.133
Distance to roads	0.003	0.002	0.065
**(C) *Passer montanus***			
(Intercept)	1.92	0.053	<0.001
w/mask-w/o mask	−0.157	0.107	0.142
Flock size	0.019	0.017	0.282
**SD (m)**	**0.047**	**0.008**	**<0.001**
Ambient noise (dcb)	0.008	0.006	0.179
Distance to roads	0.004	0.002	0.058

## Data Availability

Not applicable.
